# Prevalence, Risk Factors, and Genetic Evolution of Rat Hepatitis E Virus in Small Mammals from Southwestern Yunnan, China

**DOI:** 10.3390/biology14121685

**Published:** 2025-11-26

**Authors:** Ze Yang, Pei-Yu Han, Jun-Ying Zhao, Wei Kong, Yun Long, Song Wu, Li-Dong Zong, Chen-Jie He, Yu-Hong Chen, Wan-Chun Cao, Bo Wang, Yun-Zhi Zhang

**Affiliations:** 1 Yunnan Key Laboratory of Screening and Research on Anti-Pathogenic Plant Resources from Western Yunnan, Key Laboratory for Cross-Border Control and Quarantine of Zoonoses in Universities of Yunnan Province, Institute of Preventive Medicine, School of Public Health, School of Basic Medical Sciences, Dali University, Dali 671000, China; yangzeyz@163.com (Z.Y.); hanpeiyu1511@gmail.com (P.-Y.H.); zhaojunying0714@163.com (J.-Y.Z.); kongwei4357@163.com (W.K.); ylong991005@163.com (Y.L.); wusong080206@163.com (S.W.); zld2019106326@163.com (L.-D.Z.); h2067718141@163.com (C.-J.H.); yhchen0103@163.com (Y.-H.C.); caowanchun010209@163.com (W.-C.C.); 2 Dali Prefecture Center for Disease Control and Prevention, Dali 671000, China; 3 Programme in Emerging Infectious Diseases, Duke-NUS Medical School, 8 College Road, Singapore 169857, Singapore; bowang@nus.edu.sg

**Keywords:** hepatitis E virus (HEV), rat HEV, HEV-C1, prevalence, risk factors, genetic evolution, small mammals, Southwestern Yunan

## Abstract

Rat hepatitis E virus (rat HEV, *Rocahepevirus* genotype C1) represents a potential zoonotic threat, but its epidemiological and evolutionary characteristics in small mammals remain poorly understood, especially in regions with complex geography. Between 2022 and 2024, we collected 818 small mammals from seven border counties and cities in Yunnan, China. Rat HEV RNA was detected by RT-PCR, risk factors were assessed using binary logistic regression, and full genomes were sequenced for phylogenetic and molecular clock analysis. The overall prevalence of rat HEV was 6.23% (51/818), with significantly higher odds observed in Gengma and Heqing counties, in oriental house rat (*Rattus tanezumi*) and Chevrieri’s field mouse (*Apodemus chevrieri*), in residential habitats, and at mid-high altitudes (all *p* < 0.001). The 51 partial genomic sequences (RdRp gene) obtained in this study clustered within *Rocahepevirus*, forming two distinct subclades associated with host species. The two complete genomes, GS188 and GS197 from *Rattus tanezumi*, were classified as subtypes C1b and C1d, respectively. Bayesian analysis estimated that GS197 diverged from a closely related *Rattus tanezumi*-derived strain around 1998, while GS188 diverged from a lineage containing shrew and human strains around 1931. These findings reveal a relatively high prevalence and substantial genetic diversity of *Rochepevirus* in southwestern Yunnan, suggesting human-influenced transmission dynamics and a potential for cross-species infection.

## Simple Summary

We investigated the prevalence, risk factor and genetic evolution of rat hepatitis E virus (rat HEV) in small mammals from southwestern China. A total of 818 animals from different habitats and elevations were analyzed to understand how the virus circulates in wildlife and which factors contribute to its transmission. Rats living near human settlements, especially in residential and farming areas, showed higher infection rates. Certain species, particularly Chevrier’s field mouse and the Asian house rat, were more likely to carry the virus. Genetic analysis indicated that rat HEV can adapt to multiple hosts and may have the potential to infect humans. These findings underscore the need for continued wildlife surveillance to reduce the risk of future zoonotic disease.

## 1. Introduction

Hepatitis E virus (HEV) is a single-stranded, positive-sense RNA virus and a major pathogen responsible for acute viral hepatitis [1,2]. HEV infection has emerged as a significant global public health challenge. A recent systematic review and meta-analysis estimated that approximately 12.47% of the global population (around 939 million) have experienced a past HEV infection, while 15 to 110 million individuals are either currently or recently infected [3]. HEV can cause both outbreaks and sporadic cases of hepatitis, typically manifesting as a self-limiting illness [4]. However, in immunosuppressed individuals, HEV infection may progress rapidly to liver cirrhosis [5]. In the general population, the mortality ranges from 0.2% to 1.0%, but it is significantly higher during pregnancy, especially in developing countries [6,7]. Moreover, HEV-associated central nervous system disorders have also been reported in infected patients [8].

HEV belongs to the family *Hepeviridae*, which the International Committee on Taxonomy of Viruses (ICTV) divided into two subfamilies: *Orthohepevirinae* and *Parahepevirinae*. The subfamily *Orthohepevirinae* includes four genera: *Paslahepevirus*, *Rocahepevirus*, *Avihepevirus*, and *Chirohepevirus*. The genus *Rocahepevirus* has been detected in a wide range of animal hosts, with rodents serving as the primary reservoirs. However, its recent identification in diverse species such as shrews, voles, brown bears, ferrets, minks, kestrels, falcons, and red foxes indicates a broad host range and considerable genetic diversity [2,9,10]. The genus currently comprises two recognized species: *Rocahepevirus ratti* (which includes HEV-C1, HEV-C2 and HEV-C3 genotypes) and *Rocahepevirus eothenomi* (accommodating the putative HEV-C4 genotype). In addition, novel putative genotypes such as HEV-C3 and HEV-C4 have been reported in Chevrier’s field mouse and Père David’s vole since 2018 [11]. Furthermore, a strain isolated from Rattus norvegicus has recently been tentatively designated as genotype HEV-C5 [12]. The rat HEV has been classified as genotype HEV-C1 [13]. It has been proposed that HEV-C1 can be further genetically segregated into four subtypes, designated HEV-C1a to HEV-C1d [14,15]. To date, *Rocahepevirus* sequences have been documented in 25 animal species across 22 countries, spanning 8 species within the family *Muridae* and 10 species within the family *Cricetidae* (order Rodentia) [16].

Rat hepatitis E virus (rat HEV), a member of the genus *Rocahepevirus*, was long believed to infect only rodents [16,17]. This view changed in 2018 when the first human case of rat HEV infection was reported in Hong Kong, China [18]. The strain isolated from a liver transplant recipient shared 99.2% genomic identity with local rat HEV strains from Norway rats (*Rattus norvegicus*). The patient’s serum reacted strongly to the rat HEV-specific capsid protein p241 but not to the human HEV antigen p239, confirming that rat HEV represents a distinct lineage [19]. This discovery overturned the assumption of species-restricted tropism of rat HEV and highlighted its potential for interspecies transmission. Since then, chronic rat HEV infections have been reported in immunocompromised patients, pediatric cases, and HIV-positive individuals in Germany, Spain, France, and other regions [20,21,22]. The global evidence continues to accumulate, with recent reports further substantiating its clinical significance. These include a chronic infection in a kidney transplant recipient from Hainan, China, in which the viral strain showed close phylogenetic relatedness to rat HEV variants circulating in local rodent populations [23]. A similar chronic hepatitis outcome was observed in a heart transplant patient in Thailand following rat HEV infection [24]. Notably, a hospital-based study in the biodiversity-rich region of Yunnan identified rat HEV in 6 of 865 general patients [25], underscoring a tangible spillover burden in areas with high human–animal interaction. Despite its growing recognition, rat HEV remains underdiagnosed due to limitations in diagnostic assays and screening protocols, which likely contribute to the underestimation of acute hepatitis cases of unknown origin [25].

Yunnan Province, located in China’s southwestern border region adjoining Myanmar, Laos, and Vietnam, is a global biodiversity hotspot and a critical hub for the cross-border spread of zoonotic viruses [26]. Its unique tropical-subtropical climate gradient and complex topography sustain high small mammal diversity (e.g., rodents and shrews). Frequent human–animal interactions (such as mixed farming practices), active livestock trade, and transboundary wildlife movement elevate HEV transmission risks for both humans and domestic animals (cattle, goats) [27]. HEV prevalence rates of 7.79–22.70% have been reported in Yunnan’s small mammals [12,28]. Infections are not limited to domestic rats (*Rattus tanezumi*, *R. norvegicus*) but extend to tree shrews (*Tupaia belangeri*) and shrews (*Soricidae*) [29], demonstrating broad viral adaptability across *Rodentia*, *Scandentia*, and *Eulipotyphla*. Considerable genotypic diversity has also been observed, including emerging genotypes such as HEV-C3 [11]. These ecological and virological features establish Yunnan as an important reservoir for HEV: high densities sustain viral circulation, multiple host species facilitate cross-species transmission, and genetic diversity enables continuous viral adaptation, creating hotspots for emerging infectious diseases [26].

To better understand the infection and evolution of rat HEV, we collected small mammals from seven counties and cities in southwestern Yunnan Province, China. Through molecular epidemiology, phylogenetic reconstruction, and evolutionary dating, this study aimed to (i) characterize prevalence patterns, (ii) assess host adaptability and cross-species potential, and (iii) elucidate genomic features and evolutionary history of rodent-derived HEV strains, thereby providing scientific evidence to guide the prevention and control of small mammal-borne HEV.

## 2. Materials and Methods

### 2.1. Ethics Statement

This research was approved by the Medical Ethics Committee of Dali University under number 2021-PZ-177. All animals were treated according to the Guidelines of Regulations for the Administration of Laboratory Animals (Decree No. 2 of the State Science and Technology Commission of the People’s Republic of China, 1988) and the Guidelines for Treating Animals Kindly from Ministry of Science and Technology of the People’s Republic of China. All efforts were made to minimize discomfort to the animals.

### 2.2. Sample Collection and Viral RNA Extraction

**Figure 1:**
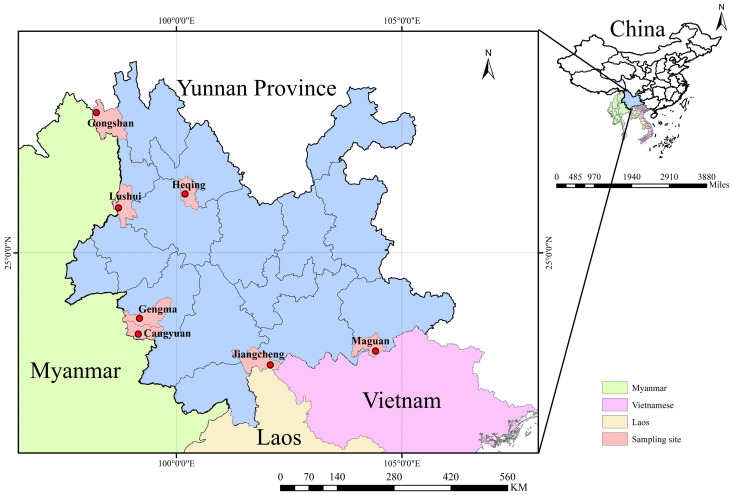
Sampling sites for small mammal specimens in Southwestern Yunnan. The map (upper right) shows the location of Yunnan Province in relation to neighboring countries (Myanmar, Laos, and Vietnam). The red-highlighted areas (lower left) mark the seven sampling sites within Yunnan Province. The blue area represents Yunnan Province, China.

From July 2022 to October 2024, small mammals were collected from residential areas, farms, shrublands, and forests across seven counties and cities in Yunnan Province, including Gongshan, Lushui, Heqing, Cangyuan, Gengma, Jiangcheng, and Maguan, spanning the province from west to south (Figure 1). Animals were trapped using baited mouse cages (20 × 12 × 10.5 cm; Xiangyun Hong Jin Mouse Cage Factory, Dali, Yunnan, China) with fresh fried dough as bait. After capture, small mammals were anesthetized using isoflurane (delivered via cotton in an induction chamber at a concentration of 1 mL per 500 mL chamber volume). Following the onset of anesthesia, they were euthanized on a warming pad to minimize distress. Tissue samples (heart, liver, spleen, lung, kidney, and rectum) were collected from all animals, aliquoted into pre-cooled 2 mL cryotubes (CORNING, Shanghai, China), stored in liquid nitrogen, and subsequently transferred to a −80 °C ultra-low temperature freezer until testing.

Species identification was performed using a morphology–molecular dual verification system. First, taxonomic experts conducted morphological classification based on external characteristics. This was followed by molecular identification through amplification and Sanger sequencing of the mitochondrial cytochrome b gene (*mt-Cytb*) [30,31,32].

Under sterile conditions, approximately 1 g of liver tissue was placed into a GeneReady Animal PIII grinding tube (Life Real, Hangzhou, China) containing 600 μL of sterile phosphate-buffered saline (PBS). Samples were homogenized using a GeneReady Ultimate homogenizer (Life Real, Guangzhou, China). A total of 300 μL of the resulting supernatant was used for RNA extraction with a commercial kit (TIANGEN, Beijing, China) on a fully automated nucleic acid extraction and purification system (BIOER, Hangzhou, China). Extracted RNA was stored at −80 °C for subsequent analysis.

### 2.3. Detection of HEV RNA

Viral RNA was screened using a semi-nested RT-PCR assay with broad-range primers targeting the RdRp region, capable of amplifying various members of the Hepeviridae family [10,33]. The amplification conditions were optimized from the original protocol. The definitive identification of rat HEV was confirmed by sequencing the obtained amplicons and subsequent phylogenetic analysis. Each PCR round was performed in a 25 μL reaction mixture. In the first round, a one-step RT-PCR kit (TIANGEN, Beijing, China) was used with 3 μL of RNA template. The thermal cycling conditions were as follows: reverse transcription at 42.0 °C for 30 min; initial denaturation at 94.0 °C for 3 min; 35 cycles of 94.0 °C for 30 s, 52.8 °C for 30 s, and 72.0 °C for 30 s; followed by a final extension at 72.0 °C for 5 min and hold at 4.0 °C. The second round was performed with Master Mix (Vazyme, Nanjing, China) using 1 μL of the first-round product as template. Cycling conditions were 94 °C for 3 min; 35 cycles of 94 °C for 30 s, 55 °C for 30 s, and 72 °C for 30 s; with a final extension at 72 °C for 5 min [33].

Amplification products were examined by agarose gel electrophoresis. Bands of the expected size were excised and purified using a gel extraction kit (OMEGA Bio-Tek, Norcross, GA, USA), and bidirectional Sanger sequencing was conducted by Sangon Biotech (Shanghai, China) Co., Ltd. Samples yielding overlapping peaks or sequencing failures were re-purified (OMEGA Bio-Tek, Norcross, GA, USA), cloned into the pGEM-T Easy Vector (TransGen Biotech, Beijing, China), and sequenced from positive clones.

### 2.4. Statistical Analysis

The prevalence of rat HEV in small mammals was calculated as the number of positive samples divided by the total number of animals tested. Associations between HEV infection status and explanatory variables were initially assessed using Pearson’s chi-square test or Fisher’s exact test, as appropriate. Variables with statistical significance (*p* < 0.05) in univariate analysis were subsequently included in a binary multivariate logistic regression model to evaluate their independent effects while controlling for potential confounders. Model fit was assessed using the Hosmer–Lemeshow test. Results were reported as adjusted odds ratios (ORs) with corresponding 95% confidence intervals (CIs). All statistical analyses were conducted using SPSS version 24.0 (IBM Corp., Armonk, NY, USA).

### 2.5. High-Throughput Sequencing and Whole Genome Acquisition

Viral RNA from positive samples was used to construct sequencing libraries, and paired-end sequencing was performed on the MGISEQ-2000 platform (MGI, Shenzhen, China). Raw data were processed through joint quality control using Trimmomatic v0.39 and FastQC v0.11.9 to remove low-quality sequences. Adapter contamination was eliminated using Cutadapt v3.4, yielding high-quality clean reads.

The processed data were aligned against a custom HEV reference database using the DIAMOND v2.0.13 algorithm (Crystal Impact GbR, Tübingen, Germany), and candidate sequences with ≥95% confidence were retained. De novo assembly was performed in parallel with SOAPdenovo2 (BGI, Shenzhen, China) and MEGAHIT v1.2.9 (HKU, Hong Kong, China), and resulting contigs were validated through BLASTn (https://blast.ncbi.nlm.nih.gov, accessed on 12 September 2025) homology searches. Final sequence correction and open reading frame (ORF) annotation were conducted in Geneious Prime v2025.0.2 (Biomatters Ltd., Auckland, New Zealand), using the reference genome as a guide.

### 2.6. Bioinformatics Analysis

Representative HEV gene sequences were retrieved from NCBI GenBank database for phylogenetic analysis. Multiple sequence alignment was performed using the MAFFT algorithm in Geneious Prime, with manual correction for reading frame shifts. A phylogenetic tree was constructed using the neighbor-joining method based on p-distances with 1000 bootstrap replicates to assess the robustness of the tree topology.

Recombination analysis was conducted using RDP4 [34]. Six algorithms, including RDP, Chimaera, BootScan, GENECONV, MaxChi, and SiScan, were applied to detect potential recombination events, and only events supported by at least four methods were retained. For each candidate event, corresponding sequences were extracted, realigned with MAFFT, and further validated in Simplot v3.5.1 (window size: 200 bp; step size: 20 bp).

The best-fit nucleotide substitution model was identified using IQ-Tree. Bayesian evolutionary inference was performed with BEASTv1.10.4 [35]. Parameters, including sampling times, substitution models (GTF+G), and molecular clock models (uncorrelated relaxed clock), were used. The Markov chain Monte Carlo (MCMC) analysis was run for 3 × 10^7^ iterations, and priors were configured using the BEAUti interface. MCMC runs were executed in BEAST to generate posterior distributions of trees. Tree Annotator was applied to discard burn-in and summarize tree statistics, while Fig Tree v1.4.4 was used for visualization, annotation, and calibration of divergence times.

## 3. Results

### 3.1. Sample Collection and HEV Detection

A total of 818 small mammals were collected, representing 17 species, 7 families, and 4 orders. Among these, the oriental house rat (*Rattus tanezumi*) comprised 35.45% (290/818), Chevrieri’s field mouse (*Apodemus chevrieri*) 24.69% (202/818), and other species collectively 39.86% (326/818). The samples included 399 females (48.78%) and 419 males (51.22%) (419/818). Notably, a high prevalence was also observed in *R. andamanensis* (1/5, 20.00%), though this estimate should be interpreted with caution due to the very small sample size. Age composition consisted of 14 juveniles (1.71%), 87 sub-adults (10.64%), and 717 adults (87.65%). Samples were obtained from four habitat types: residential areas (9.90%, 81/818), farming areas (31.42%, 257/818), shrublands (26.04%, 267/818), and forest areas (26.04%, 213/818). By elevation, 46.09% (377/818) were collected at 0–1499 m, 27.87% (228/818) at 1500–2999 m, and 26.04% (213/818) above 3000 m. The overall prevalence of rat HEV was 6.23% (51/818). Positive samples were detected in the oriental house rat (*R.* *tanezumi*), Chevrieri’s field mouse (*A. chevrieri*), the large Chinese vole (*Eothenomys miletus*) and the black-toothedged rat (*R. andamanensis*) (Table 1).

### 3.2. Analysis of Factors Influencing Rat HEV Infection in Small Mammals

Among the 818 collected small mammals tested, rat HEV prevalence varied significantly across sampling locations (χ^2^ = 90.29, *p* < 0.001). By host species, prevalence was highest in *A.* *chevrieri* (12.87%, 26/202), followed by *R. tanezumi* (7.93%, 23/290), and other species (0.61%, 2/326), with significant interspecies differences (χ^2^ = 34.27, *p* < 0.001). Age group analysis showed prevalence rates of 14.29% (2/14) in juveniles, 3.45% (3/87) in sub-adults, and 6.42% (46/717) in adults; however, these differences were not statistically significant (χ^2^ = 2.75, *p* = 0.25). Habitat distribution revealed marked variation: 20.99% (17/81) in residential areas, 10.51% (27/257) in farming areas, 2.62% (7/267) in shrublands, and 0.00% (0/213) in forest areas (χ^2^ = 58.30, *p* < 0.001). Pairwise comparisons confirmed significant differences among habitats, with residential areas showing the highest prevalence. Prevalence also differed by elevation: 5.31% (20/377) at 0–1499 m, 13.60% (31/228) at 1500–2999 m, and 0.00% (0/213) at ≥3000 m (χ^2^ = 38.85, *p* < 0.001). The highest prevalence occurred at mid-elevation (1500–2999 m) (Table 2).

Binary logistic regression, incorporating significant factors from univariate analysis (sampling location, species, habitat, and elevation), identified the following independent associations: the prevalence of rat HEV in small mammals from Gengma County and Heqing County was significantly higher than that in the reference group (Cangyuan County), being 8.14 times higher (95% CI: 2.90–22.87, *p* < 0.001) and 4.93 times higher (95% CI: 1.97–12.32, *p* < 0.001), respectively; the prevalence of rat HEV in *R. tanezumiand* and *A. chevrieri* were significantly higher, reaching 14.00 times (95% CI: 3.27–59.89, *p* < 0.001) and 24.14 times (95% CI: 5.67–102.88, *p* < 0.001), compared with other species; the prevalence of rat HEV in residential areas was significantly higher than in shrublands, with an odds ratio (OR) of 9.87 (95% CI: 3.93–24.80, *p* < 0.001); the prevalence of rat HEV at mid-elevation (1500–2999 m) was significantly higher than at low elevation (0–1499 m), with an OR of 2.81 (95% CI: 1.56–5.06, *p* < 0.001) (Table 2).

### 3.3. Phylogenetic Analysis and Co-Evolution with Hosts of Rat HEV

**Figure 2:**
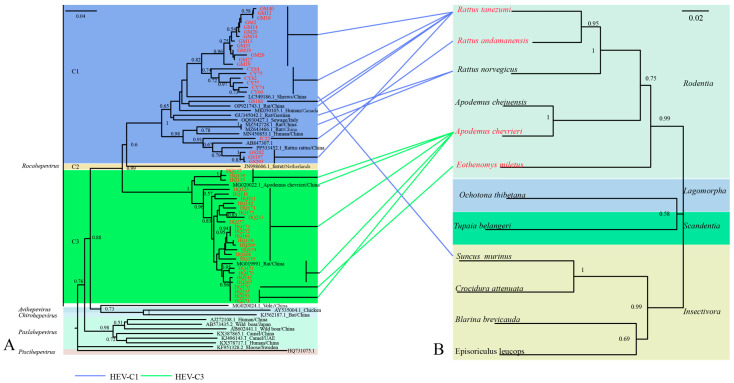
Phylogenetic analysis of rat HEV strains and their small mammal hosts. (**A**) Phylogenetic tree of 51 rat HEV strains obtained in this study, constructed using the neighbor-joining method based on partial RdRp sequences. Strains highlighted in red indicate rat HEV sequences from small mammals identified in this study. (**B**) Phylogenetic tree of small mammal hosts based on the mitochondrial cytochrome b (*mt-Cytb*) gene.

The 51 rat HEV strains obtained in this study (GenBank accession numbers: PX233014–PX233064), based on the sequencing of a 338 bp RdRp gene fragment, exhibited nucleotide (nt) sequence homologies ranging from 64.69% to 100.00% (Figure S1). Phylogenetic analysis revealed that all strains clustered within the *Rocahepevirus* lineage (Figure 2). Phylogenetic analysis revealed a clear division of the sequences into two clades: HEV-C1 and HEV-C3. HEV-C1 contained 24 sequences, among which were three strains (GS197, GS202, and GS203 from *R. tanezumi*) clustered with strain JC22 from *R. nitidus* in Yunnan and a human HEV strain from Hong Kong (GenBank accession no. MN450544). The remaining 19 strains from *R. tanezumi* were closely related to a shrew-derived HEV strain from Guangdong (GenBank accession no. LC549186) and a human strain from Canada (GenBank accession no. MK050105) [36]. HEV-C3 comprised 27 sequences with nt identities of 72.41–100.00%, including one strain from *E. miletus* and 26 strains from *A. chevrieri*, all detected in Heqing County. These clustered closely with rat HEV strains from *A. chevrieri* in Lijiang, Yunnan (GenBank accession no. MG020022).

Comparison of HEV and host phylogenetic trees demonstrated greater identity among strains originating from the same host species. For example, the 27 *A. chevrieri* strains from Dali Prefecture were closely related to *A. chevrieri*-derived strains from Lijiang City (GenBank accession nos. MG020022 and MG019991). Similarly, strains GM10 and CY75, from *R. tanezumi* in Gengma and Cangyuan Counties, respectively, formed a tight cluster, while GS188 from Gongshan County resided within the same major clade. Nevertheless, exceptions to this virus–host co-clustering pattern were observed. A notable example is the strain LC549186, which was isolated from the insectivorous shrew (*Suncus murinus*) but clusters within a clade predominantly composed of strains from rodent hosts, suggesting a potential cross-species transmission event (Figure 2).

### 3.4. Genomic Structure and Similarity Comparison of Two Full-Length Rat HEV Sequences from Rattus tanezumi

Using high-throughput sequencing, we obtained two full-length rat HEV genomes from *R. tanezumi* in Gongshan County, Yunnan Province: GS188 (GenBank accession no. PX233012, 7023 bp) and GS197 (GenBank accession no. PX233013, 6971 bp). Both genomes contained the canonical ORF1–ORF3, with GS188 additionally harboring a complete ORF4 [17]. Genomic alignment revealed 76.94% nt identity between GS188 and GS197. Phylogenetic analysis showed that GS188 shared 46.13–83.20% nt identity with other HEV sequences encompassing all major genotypes, with the highest identity (76.94–83.20%) observed specifically with HEV-C1 strains. Likewise, GS197 exhibited 46.80–94.40% nt identity with rat HEV strains in GenBank, also showing its closest relationship (75.64–94.40%) with HEV-C1.

**Figure 3:**
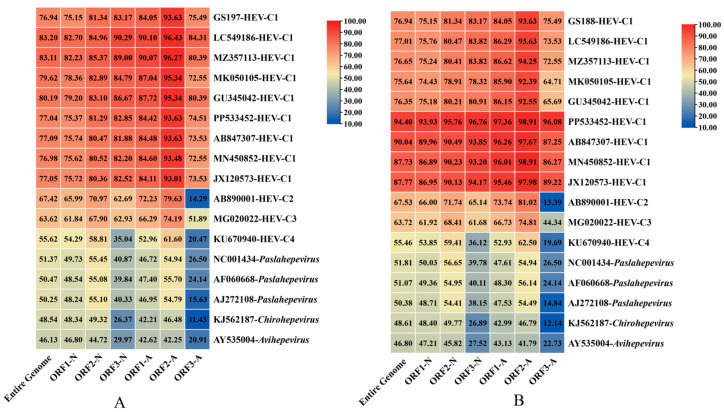
Comparison of nucleotide and amino acid sequence identity between the rat HEV strains and reference genomes. (**A**) Nucleotide and amino acid identity (%) of GS188 compared with representative HEV genomes. (**B**) Nucleotide and amino acid identity (%) of GS197 compared with representative HEV genomes.

For ORF identities, both genomes demonstrated higher nt and amino acid (aa) sequence identity with HEV-C1 strains than with other HEV genotypes, supporting their classification within the HEV-C1 lineage. Comparative analysis further revealed that, relative to other HEV-C1 strains, ORF2 of both genomes exhibited greater nt conservation than ORF1 and ORF3. Notably, ORF1 and ORF2 followed a pattern where aa identity exceeded nucleotide identity, whereas the reverse was true for ORF3, a phenomenon that has been noted in other HEV studies and may reflect differing evolutionary pressures across the genome [37] (Figure 3).

**Figure 4:**
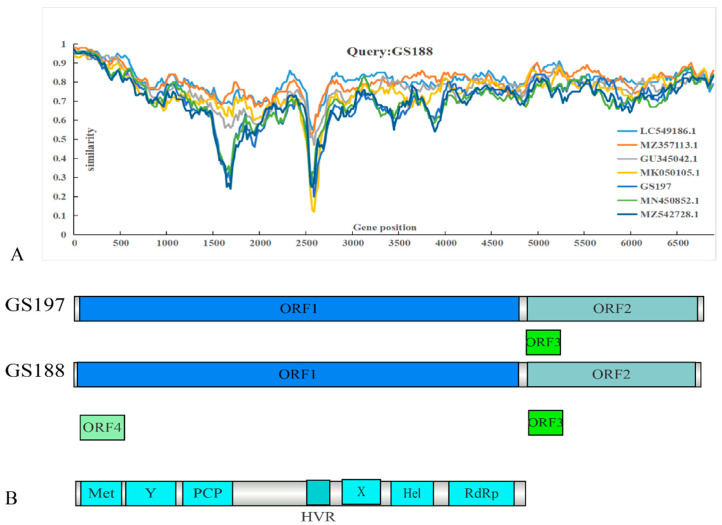
Genomic similarity and structural analysis of two rat HEV strains derived from *Rattus tanezumi*. (**A**) SimPlot analysis using GS188 as the query strain. The *y*-axis indicates nucleotide similarity, and the *x*-axis represents nucleotide position. (**B**) Full-length genomic structures and nucleotide sequences of GS188 and GS197, showing the coding regions of ORF1, ORF2, ORF3, and ORF4.

Recombination analysis using RDP4 did not reveal significant recombination events between the two rat HEV strains obtained in this study and other HEV strains. A similarity comparison was conducted using representative full-length genomes alongside the two *R. tanezumi*-derived rat HEV strains from this study (Figure 4). GS188 exhibited relatively low identity with other sequences in ORF1. Beyond the reduced identity in the hypervariable region (HVR) [38], GS188 showed a marked decrease in identity with two human-derived HEV strains from Hong Kong and the *R. tanezumi*-derived GS197 within the 1500–2000 bp segment of ORF1. In contrast, no significant decrease in identity was observed when compared to the *R. norvegicus*-derived strains from Sichuan, China (GenBank accession no. MZ357113), and Germany (GenBank accession no. GU345042), or the shrew-derived strain from Guangdong, China (GenBank accession no. LC549186).

### 3.5. Phylogenetic Analysis of Two Rat HEV Strains from R. tanezumi

**Figure 5:**
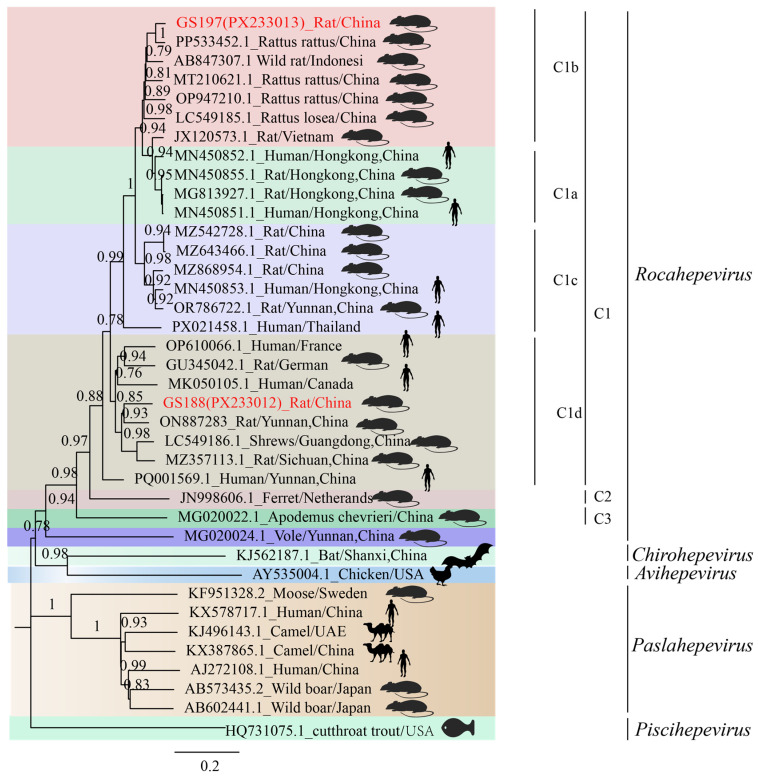
Phylogenetic analysis of *Rattus tanezumi*-derived HEV strains based on full-genome sequences. Sequences highlighted in red represent the full-length HEV strains obtained in this study.

Phylogenetic analysis based on the full-genome sequences confirmed that both GS197 and GS188 belong to HEV-C1. According to previous studies, HEV-C1 can be further divided into four subtypes (HEV-C1a, C1b, C1c, and C1d), with GS197 and GS188 falling into different subtypes (Figure 5). GS197, derived from *R. tanezumi* in Gongshan County, Yunnan Province, formed a strongly supported clade with rat HEV strain (GenBank accession no. PP533452) from *R. rattus* in China. This clade, together with rat HEV strain (GenBank accession no. MT210621) isolated from *R. norvegicus* in Wenzhou, Zhejiang, China, and rat HEV strain (GenBank accession no. LC549185) isolated from *R. losea* in Zhanjiang, Guangdong, China, belongs to HEV-C1b. In contrast, BLASTn analysis of GS188 showed the highest identity (83.51%) with HEV strain S1129 (GenBank accession no. LC549186) from shrews in Guangdong, China. In the full-genome phylogenetic tree, GS188 clustered within the HEV-C1d branch, together with the *R. norvegicus*-derived HEV strain from Sichuan (GenBank accession no. MZ357113), the shrew-derived HEV strain from Guangdong (GenBank accession no. LC549186), and a human-derived HEV strain from Canada (GenBank accession no. MK050105), highlighting its close evolutionary relationship with strains from multiple host species.

### 3.6. Temporal Evolution of Two Rat HEV Strains from R. tanezumi

**Figure 6:**
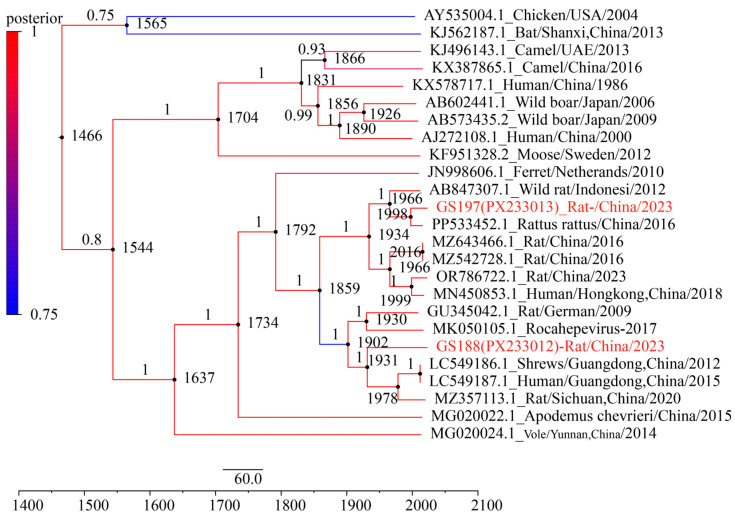
Bayesian maximum clade credibility tree of *Rattus tanezumi*-derived HEV strains based on ORF2 gene sequences, showing estimated divergence times. The tree was generated using BEAST v1.10.4 under an uncorrelated relaxed molecular clock model. The maximum clade credibility tree was inferred using BEAST under the GTR+G model. Sequences highlighted in red correspond to strains obtained in this study.

Under an uncorrelated log-normal relaxed molecular clock model, the Bayesian Skyline analysis estimated the mean evolutionary rate of the ORF2 gene to be 9.60 × 10^−4^ nt substitutions per site per year (95% Highest Posterior Density interval (HPD): 7.68 × 10^−4^–1.13 × 10^−3^). The divergence time between GS197 and another *R. tanezumi*-derived strain from Yunnan (GenBank accession no. PP533452) was estimated to be around 1998 (95% HPD: 1983–2003). In contrast, GS188 diverged earlier from other HEV strains, with an estimated divergence of approximately 1931 (95% HPD: 1872–1960) from HEV strain S1129 (GenBank accession no. LC549186) from the musk shrew (*Suncus murinus*) in Guangdong, China, and HEV strain 2PE-REP-1 (GenBank accession no. MZ375113) from the red-tailed toad-headed lizard (*Phrynocephalus erythrurus*) in Qinghai–Tibetan Plateau of China. Furthermore, the divergence of GS188 from HEV strain R63 (GenBank accession no. GU345042) from *R. norvegicus* in Germany and a human-derived HEV strain 17/1683 (GenBank accession no. MK050105) from Canada was estimated at around 1902 (95% HPD: 1885–1935) (Figure 6). These findings indicate that GS188 represents a more ancient lineage of rat HEV, suggesting long-term evolution and potential cross-species transmission over the past century.

## 4. Discussion

In this study, the overall prevalence of rat HEV in small mammals was 6.23% (51/818), which is consistent with rates reported in Yunnan and Hubei Provinces of China, as well as in Canada [28,36,39], yet it differs from other reports [12,40]. These variations highlight the influence of geographical distribution, host community structure, and methodological differences on the observed prevalence of rat HEV. The prevalence was marked higher in *A. chevrieri* (12.87%) and *R. tanezumi* (7.93%) compared with other species (0.61%, *p* < 0.001). Interestingly, the high prevalence we found in the Muridae family was similar to that detected in *Rattus norvegicus*, *Rattus rattus* and *Mus musculus* populations from different European countries [40,41]. These five species belong to the Muridae family and may share habitat and resources, so cross-species transmission of rat HEV seems plausible. More importantly, as synanthropic species (closely associated with human environments), their concentration in residential and farming areas likely amplifies transmission opportunities, a point we will elaborate on in the following discussion of ecological drivers.

Prevalence patterns also showed a strong gradient by habitat and elevation. Rat HEV was most frequent in residential areas (20.99%), followed by farming areas (10.51%), shrublands (2.62%), and forests (0.00%, *p* < 0.001), indicating that human-associated environments drive viral amplification [42]. This is consistent with previous work showing higher viral diversity in rodents habituating disturbed or anthropogenically modified habitats [26]. Residential areas provide abundant food sources (e.g., household waste), shelter (e.g., building crevices), and a stable microclimate, conditions that support dense rodent populations and increase opportunities for viral transmission [43]. Surveillance studies have shown high genetic similarity between HEV strains in sewage and those detected in local rodents [44]. This is further supported by recent findings of long-term rat HEV circulation in wild rat populations of a large metropolis, collectively suggesting sustained viral transmission at the human–wildlife interface and highlighting the successful adaptation of rat HEV to anthropized environments [45]. Moreover, pig farms frequently located near human settlements may facilitate cross-species transmission, as experimental studies demonstrate that rat HEV can infect pigs [46], and rat HEV has been detected in European pig farms [47]. Such a “pig–rat–human” transmission chain could serve as a bridge for zoonotic spillover [48].

The variation in infection rates across different habitats was further evidenced by a distinct altitudinal prevalence pattern: the mid-elevation zone (1500–2999 m) had the highest prevalence (13.60%), which was significantly greater than that in the low-elevation (0–1499 m; 5.31%) and high-elevation (≥3000 m; 0%) zones (*p* < 0.001). Mid-altitude zones host the densest human settlements and most farming activity, reinforcing the role of anthropogenic factors in shaping viral transmission risk. By contrast, high-altitude regions are relatively pristine, likely limiting rodent–human contact and viral circulation. While previous studies suggest altitude influences viral diversity in wildlife, the mechanisms remain poorly understood and warrant further investigation [49,50]. Overall, the elevated prevalence in residential and farming habitats underscores that small mammals adapting to human-modified landscapes are key amplifiers of rat HEV, representing hotspots for zoonotic risk [51].

Phylogenetic analysis confirmed that all 51 HEV sequences clustered within the rat HEV clade, with clear evidence of genetic diversity, geographic clustering, and cross-species associations. Twenty-seven strains from *A. chevrieri* in Heqing County, Dali Prefecture, clustered with strains from *A. chevrieri*-derived strains from Lijiang, suggesting long-term virus–host coexistence and lineage stability [28]. By contrast, rat HEV strains GS197, GS202, and GS203 from *R. tanezumi* in Gongshan County clustered with JC22 from *R. nitidus* from the same location and formed a branch adjacent to a human-derived strain from Hong Kong [19]. Notably, GS197 and GS188 from *R. tanezumi* also showed close genetic affinity to strains from shrews in Guangdong and human cases in Canada, as well as to *R. norvegicus* strains from Germany. These findings illustrate a broad host range, intercontinental spread potential, and phylogenetic connections between rodent- and human-derived HEV strains. Together, they provide molecular evidence that rat HEV is not restricted to a single host or region but circulates across multiple mammalian species with clear zoonotic potential [52].

Molecular clock analysis based on the ORF2 gene provided preliminary insights into the evolutionary history of *Rocahepevirus* and its major lineages. Bayesian analysis estimated that the most recent common ancestor of *Rocahepevirus* emerged approximately 1637 years ago (95% HPD: 1135–1537), corresponding to roughly the 4th to 16th centuries. This suggests a deep evolutionary history of *Rocahepevirus* within populations. The divergence of the lineage containing GS188 (along with related strains from a German rat and a Canadian human case) from other related lineages occurred earlier, around 1902–1931 (approximately 93 to 122 years ago). Our molecular clock estimates reveal a key trend: the major HEV-C1 lineages currently circulating in *R. tanezumi* populations in Yunnan, along with clades genetically related to certain human-derived isolates, represent relatively recent divergence events that likely occurred within the past one to two centuries. This time frame aligns with a period of significant global socio-ecological transformation, including accelerated urbanization, agricultural expansion, habitat fragmentation, and increased human mobility [51]. These anthropogenic changes have disrupted natural habitats and altered host animal behaviors, likely facilitating the spatial spread of the virus and increasing opportunities for interspecies contact.

## 5. Conclusions

Our study demonstrates a strong association between human-altered landscapes (particularly residential areas and mid-elevation zones in southwestern Yunnan) and high prevalence of rat HEV in small mammal populations, suggesting that human activities likely play a key role in facilitating the virus’s persistence and transmission. Rat HEV in this region exhibits substantial genetic diversity and establishes limited but notable cross-species and cross-regional phylogenetic connections, underscoring the need for continued surveillance of its zoonotic potential. These findings highlight the importance of continuous rat HEV surveillance, strengthened environmental hygiene management in human settlements, and targeted control of specific reservoir hosts to mitigate the risk of viral emergence and spillover.

## Figures and Tables

**Table 1:** Detection of hepatitis E virus in small mammals from Southwestern Yunnan.

Order	Family	Species	Composition Ratio (%)	Infection Rate (%)
*Rodentia*	*Muridae*	*Rattus tanezumi*	35.45 (290/818)	7.93 (23/290)
		*Apodemus chevrieri*	24.69 (202/818)	12.87 (26/202)
		*Apodemus draco*	1.83 (15/818)	0.00 (0/15)
		*Niviventer andersoni*	1.71 (14/818)	0.00 (0/14)
		*Rattus norvegicus*	1.71 (14/818)	0.00 (0/14)
		*Rattus rattus*	0.86 (7/818)	0.00 (0/7)
		*Rattus andanmanensis*	0.61 (5/818)	20.00 (1/5)
		*Rattus sikkimensis*	0.61 (5/818)	0.00 (0/5)
	*Cricetidae*	*Eothenomys miletus*	14.67 (120/818)	0.83 (1/120)
	*Sciuridae*	*Callosciurus erythraeus*	0.37 (3/818)	0.00 (0/3)
*Insectivora*	*Soricidae*	*Suncus murinus*	4.89 (40/818)	0.00 (0/40)
		*Anourosorex squamipes*	4.16 (34/818)	0.00 (0/34)
		*Crocidura attenuata*	2.69 (22/818)	0.00 (0/22)
		*Episoriculus leucops*	1.71 (14/818)	0.00 (0/14)
	*Erinaceidae*	*Hylomys suillus*	2.08 (17/818)	0.00 (0/17)
*Scandentia*	*Tupaiidae*	*Tupaia belangeri*	0.86 (7/818)	0.00 (0/7)
*Lagomorpha*	*Ochotonidae*	*Ochotona thibetana*	1.10 (9/818)	0.00 (0/9)
Total			100 (818/818)	6.23 (51/818)

**Table 2:** Logistic regression analysis of rat HEV prevalence in small mammals from Southwestern Yunnan.

Variable	Category	Composition Ratio (%)	Infection Rate (%)	χ^2^	*p* (Uni.) ^a^	OR (95%CI)	*p* (Multi.) ^b^
Locations	Cangyuan	17.73	4.14	90.29	<0.001	1.00 (Reference)	
	Gengma	6.11	26			8.14 (2.90–22.87)	<0.001
	Gongshan	25.43	1.92			0.45 (0.13–1.64)	0.228
	Heqing	18.83	17.53			4.93 (1.97–12.32)	<0.001
	Jiangcheng	6.36	1.92			0.45 (0.05–3.87)	0.47
	Lushui	6.36	0			0	0.991
	Maguan	6.11	0			0	0.985
Species	Others	39.85	0.61	35.27	<0.001	1.00 (Reference)	
	*Rattus tanezumi*	35.45	7.93			14.00 (3.27–59.89)	<0.001
	*Apodemus chevrieri*	24.69	12.87			24.14 (5.67–102.88)	<0.001
Sex	Female	48.78	6.78	0.36	0.55	NA ^c^	NA ^c^
	Male	51.1	5.76				
Age	Childhood	1.71	14.29	2.75	0.25	NA ^c^	NA ^c^
	Subadult	10.64	3.45				
	Adult	87.65	6.42				
Landscape	Shrubbery area	26.04	20.99	58.3	<0.001	1.00 (Reference)	
	Cultivated area	31.42	10.51			4.36 (1.86–10.20)	<0.001
	Residential area	9.9	2.62			9.87 (3.93–24.80)	<0.001
	Forest area	26.04	0			0	0.983
Altitude (m)	0–1499	46.09	5.31	38.85	<0.001	1.00 (Reference)	
	1500–2999	27.87	4.88			2.81 (1.56–5.06)	<0.001
	3000-	26.04	7.52			0	0.982

^a^ *p* (Uni.): Univariate analysis—Pearson’s chi-square test or Fisher’s exact test. ^b^ *p* (Multi.): Multivariate analysis—binary logistic regression model. ^c^ NA: Indicates that the factor was not included in the logistic regression analysis due to a *p*-value > 0.05 in univariate analysis.

## Data Availability

The datasets used in this study can be accessed by contacting the corresponding author in a reasonable manner. All sequences referenced in this article are retrievable from the NCBI database (https://www.ncbi.nlm.nih.gov, accessed on 1 August 2025).

## Supplementary Materials

The following supporting information can be downloaded at https://www.mdpi.com/article/10.3390/biology14121685/s1, Figure S1: Identity comparison of nucleotide and amino acid sequences of the rat HEV RdRp fragment in small mammals from this study. The upper right triangle of the matrix represents amino acid sequence identity (%); the lower left triangle represents nucleotide sequence identity (%).
