# Supplementary material for: Prevalence, Risk Factors, and Genetic Evolution of Rat Hepatitis E Virus in Small Mammals from Southwestern Yunnan, China
Source: Biology (Basel). 2025 Nov 26;14(12):1685. doi: 10.3390/biology14121685 (PMC12730198; doi:10.3390/biology14121685)
Supplement: Supplementary file 1 [file biology-14-01685-s001.zip › biology-3923654-supplementary.pdf]

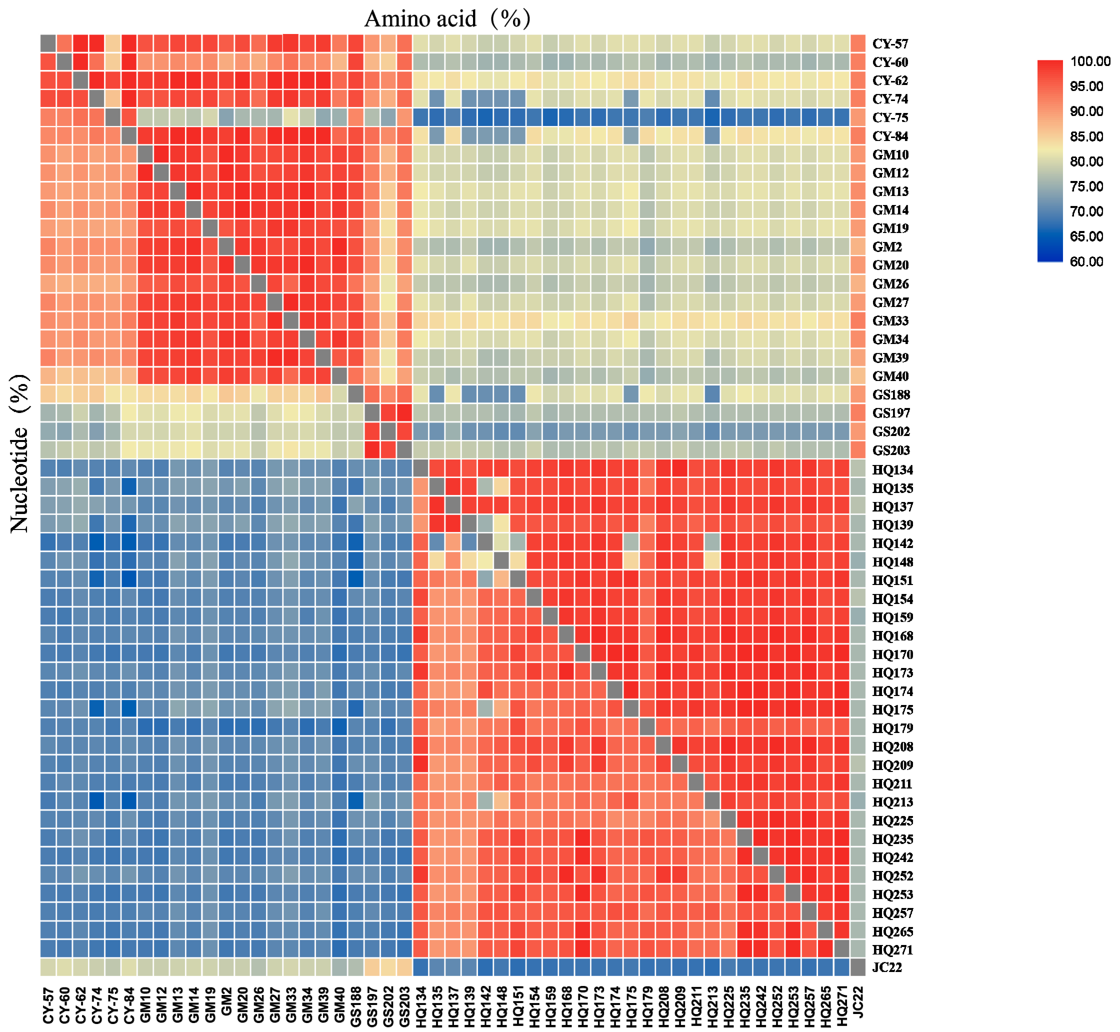

Figure S1. Identity comparison of nucleotide and amino acid sequences of the Rat HEV RdRp fragment in small mammals from this study. The upper right triangle of the matrix represents amino acid sequence identity (%); the lower left triangle represents nucleotide sequence identity (%).
